# Arrhythmogenic cardiomyopathy. Patterns of ventricular involvement using cardiac magnetic resonance

**DOI:** 10.1186/1532-429X-13-S1-P268

**Published:** 2011-02-02

**Authors:** Begoña M Igual, Zorio G Esther, Maceira G Alicia, Estornell E Jordi, Lpoez L Pilar, Monmeneum M Jose Vicente, Eetelles M Fernando, Lucas P Almudena

**Affiliations:** 1 Eresa, Valencia, Spain; 2 Familiar Sudden Death Unit,.Hospital La Fe., Valencia, Spain

## Introduction

Recently, biventricular (ABVC) and left dominant arrhythmogenic cardiomyopathy (LDAC) had been included in the spectrum of arrhythmogenic cardiomyopathy (AC).

## Purpose

The aim of the study was to describe using cardiac magnetic resonance, the patterns of ventricular involvement as well as of late gadolinium enhancement (LGE).

## Methods

Medical records and databases from 3 hospitals were reviewed (2006-2010) in order to obtain data of patients with AC. Diagnosis of classic ARVC and BVAC was based on Task Force criteria (TFC). LDAC was diagnosed if LGE was present along with a positive family history. Now all our LDAC patients had genetic diagnosis and meet current TFC.

## Results

26 consecutive patients were included (40+16yrs, 16 males). Diagnoses were: LDAC in 8 patients (30%),All of them had desmoplakin disease and 3 combined desmoplakin and desmocolin disease. BVAC in 9 (35%) and ARVC in 9 (35%). Right ventricular involvement was present in 19 patients (73%). Among them, 13 patients (50%) had ventricular volumes above the upper limit of normality and 6 (23%) had mild involvement with wall motion abnormalities and microaneurysms. Also, 11 patients (42%) showed LGE in the right ventricle and 25 patients (96%) in the left ventricle. LGE was more frequent in the inferior and inferolateral walls (17 and 16 patients, 65 and 62%) while the septum was seldom affected (7 patients, 27%). Usually, LGE was subepicardial (12 patients, 46%), but transmural (5 patients, 19%) and intramyocardial (3 patients, 12%) LGE were also observed. Left ventricle systolic dysfunction (LVEF<55%) was observed in 15 patients, (57 %,),2 patients (7%) with LVEF< 40% and left ventricular dilatation (LVEDVi>98ml/m2) in 3 patients, (11%)

## Conclusions

1.LV involvement is a frequent finding in AC 2. The most frequent abnormality is LGE in the left ventricle and the least, left ventricular dilatation 3.LGE was more frequently subepicardial and located in the inferior and inferolateral walls.

